# Lipid-based nutritional supplement impact on energy intake, appetite, glucose and insulin levels in under-weight pregnant and lactating women with preeclampsia

**DOI:** 10.1042/BSR20231344

**Published:** 2024-01-25

**Authors:** Nabila Sher Mohammad, Rubina Nazli, Sadia Fatima, Fozia Fozia, Hafza Zafar, Mashal Zafar, Zarghuna Zafar, Warda Khan, Mahmoud M.A. Abulmeaty, Dara Aldisi, Juan E. Andrade Laborde, Mourad A.M. Aboul-Soud

**Affiliations:** 1 Department of Biochemistry, Institute of Basic Medical Science, Khyber Medical University, Peshawar 25000, Pakistan; 2 Department of Biochemistry, KMU Institute of Dental Science, Kohat 26000, Khyber Pakhtunkhwa, Pakistan; 3 Institute of Physical Medicine and Rehabilitation, Khyber Medical University, Peshawar 25000, Pakistan; 4 Rehman Dental Collage, Peshawar 25000, Pakistan; 5 Department of Community Health Sciences, College of Applied Medical Sciences, King Saud University, Riyadh 11362, Kingdom of Saudi Arabia; 6 Department of Food Science and Human Nutrition, University of Florida, 110370 Gainesville, FL, U.S.A.; 7 Department of Clinical, Laboratory Sciences, College of Applied Medical Sciences, King Saud University, Riyadh 11433, Saudi Arabia

**Keywords:** energy intake, Glucose, insulin, LNS-PLW, preeclampsia

## Abstract

Objective: The objective of the study is to investigate the response of nutritional supplement (LNS-PLW) on appetite score, energy intake, insulin and glucose levels in preeclamptic women.

Design and participiants: Sixty under-weight preeclamptic primigravida were divided into two groups randomly and provided LNS-PLW/placebo in the fasted state. Blood samples were collected at fasting state, after 30 min of supplementation, ‘*ad libitum* buffet’ breakfast and lunch for glucose and insulin levels.

Results: Total energy intake was higher significantly in the LNS-PLW group, although during breakfast it was significantly reduced. The insulin and glucose concentration was significantly increased after 30 min of supplementation in the LNS-PLW group.

Conclusion: Intake of the LNS-PLW by preeclamptic women had short-term suppression on subsequent meal but improved total energy intake during trial.

## Introduction

Pregnancy is followed by a series of regular changes in the maternal body [1]. If any impairment occurs, serious complications like preeclampsia may present and become a threat to maternal and fetal health [2]. Preeclampsia is a multisystem disorder characterized by high blood pressure, proteinuria and oedema in previously normotensive women [3]. It is a pregnancy-specific disease affected 3–5% of women globally and is documented as one of the major causes of maternal and neonatal morbidity, mortality and stillbirths [4–6]. In our country, nearly 19% of maternal mortality is credited to preeclampsia [4,7]. Due to unreliable prognosis and diagnosis of preeclampsia, its management is a challenging task [8,9].

Inadequate diet has been documented as an association with poor maternal outcomes (increased risk of preeclampsia, gestational diabetes and extreme gestational weight gain), negative birth outcomes (premature and low birth weight), and childhood and adult health outcomes (obesity and chronic heart disease) [10,11]. Pregnant women with a low socioeconomic status faced an additional challenge to maintaining a healthy diet due to a lack of money, information and skills to make nutritional modifications [10].

Under nutrition in childbearing-age women of Pakistan has been identified as a serious issue, 50% of women during pregnancy were found anaemic and most of them were found macro and micronutrient deficient [12]. Due to the additional stress of the developing placenta, foetus and maternal metabolism, pregnant women are more susceptible to this deficits [13]. Future complications could be decreased with the antenatal and postnatal follow-up that includes dietary counselling, lifestyle changes and nutritional supplements [14]. LNS-PLW is a special lipid-based dietary food supplement of the World Food Programme (WFP), designed to complement pregnant and lactating women’s diets as a part of a nutritional programme for underdeveloped countries [15]. The product is intended to be eaten directly from the sachet without dilution, mixing or cooking to prevent contamination. One sachet contains 75 g of a high-energy, nutrient-dense formula consisting of several micronutrients (i.e., K, I, Cu, Fe, Se, Zn, Ca, P, Mg, B complex and vitamins A, C, D, E and K) and macronutrients (carbohydrate, protein and fat) providing 400 kcal per sachet as shown in Table 1.

**Table 1:** Nutritional value chemical composition and of LNS-PLW

Total energy		400 kcal	
Nutrients	Value	Nutrients	Value
Carbohydrate	35.4 g	Cholecalciferiol (vitamin D)	11.2 mcg
Protein	10.5 g	Tocoferol acetate (vitamin E)	12 mg
Fat	24 g	Phytomenadione (vitamin K)	20.2 mcg
Retinol (vitamin A)	412 mcg	Ca	400 mg
Thiamine (vitamin B1)	0.75 mg	Cu	1.0 mg
Ribofloin (vitamin B2)	1.57 mg	I	75 mcg
Niacin (vitamin B3)	9.75 mg	Fe	7.5 mg
Pantothenic acid (vitamin B5)	3.0 mg	Mg	112 mg
Pyridoxine (vitamin B6)	1.35 mg	Mn	0.9 mg
Biotin (vitamin B7)	45 mcg	P	337 mg
Folates (vitamin B9)	247 mcg	K	675 mg
Cobalamine (vitamin B12)	2.0 mcg	Se	15 mcg
Ascorbate (vitamin C)	45 mg	Zn	8.2 mg

Nutritional supplements are often recommended to boost body weight, food and energy intake resulting in improved health outcome among malnourished [16–18]. Although these high-energy supplements increase daily energy intake, their net beneficial effects might not be achieved as these also increase satiety post-consumption [19,20]. This may be due to partially displacing food and energy intake in habitual meals due to a reduction in hunger and increased satiety [19,20].

The beneficial role of multiple micronutrient supplements on cardiovascular health and hypertension in normal pregnancy, underweight young girls and children is reported [17,18,21]. However, few studies reported that energy intake reduces after supplementation in underweight young girls and children [16–18]. Insufficient evidence is available on the short-term effect of lipid based nutritional supplements intervention on energy intake, appetite score and metabolic biomarkers like glucose and insulin levels in women with preeclampsia [13].

The study aimed to examine the short-term effect of LNS on appetite score, glycaemic and insulin response, and the subsequent energy intake among preeclamptic women.

## Material and method

### Background

A randomized controlled trial (RCT) was conducted in the Post Graduate Medical Institute Hayatabad Medical Complex (PGMI, HMC), Post Graduate Medical Institute Lady Reading Hospital Peshawar (PGMI, LRH) and Civil Matta Hospital Swat, Khyber Pakhtunkhwa, Pakistan. The trial got approval from the institutional committee (DIR/KMU-AS&RB/EN/000527) and ethical approval (DIR/KMU = EB/EN/000314) from Khyber Medical University Peshawar on 27 October, 2016. This trial got registered with ISRCTN UK number (international standard randomised control trial number of the United Kingdom (ISRCTN15485068, April 2018; https://doi.org/10.1186/ISRCTN15485068).

### Study design

#### Inclusion criteria/exclusion criteria

According to the inclusion criteria of this study, the study population includes underweight preeclamptic primigravida with BMI at the first antenatal visit lower than required BMI for the respective gestational age retrospectively from their antenatal record. Participants were non-smokers and were not following any special diet or nutritional supplement. The exclusion criteria included women having a history of diabetes mellitus, hypertension, renal, liver diseases and allergic to ingredients (i.e., dairy, pulses and lecithin) in the LNS-PLW.

#### Sampling technique

**Figure 1:**
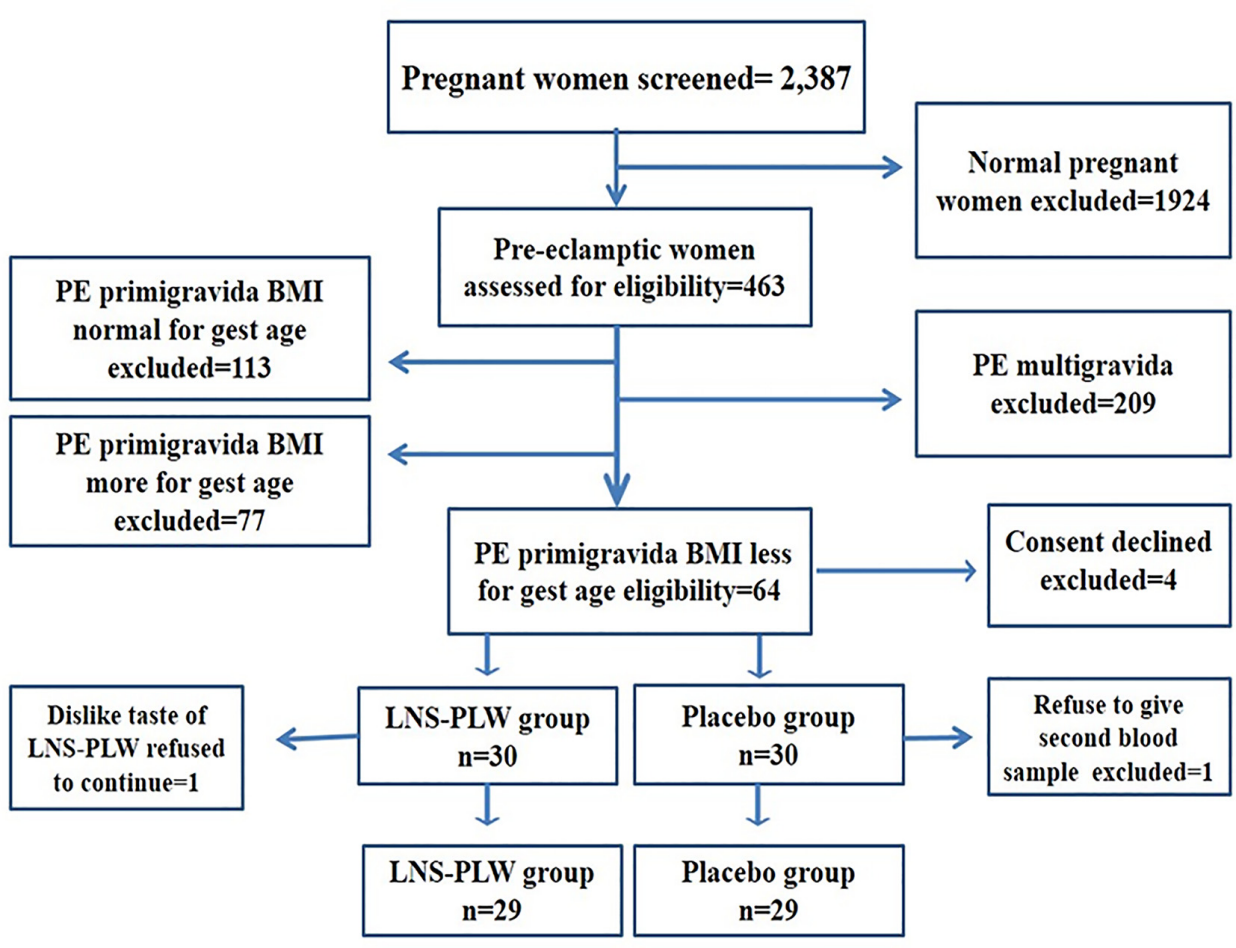
Flow chart of participant screening and recruitment in study

We screened 2387 pregnant women in the tertiary care antenatal clinics of Peshawar and Matta Swat. Out of 2387 pregnant women, 463 were found preeclamptic while 1924 were normal pregnant women so excluded. After screening based on our selection criteria, 209 multigravida women with preeclampsia were excluded. The antenatal records of the remaining preeclamptic primigravida were screened retrospectively for weight and gestational age on their first antenatal visits. The preeclamptic primigravida with normal BMI according to their gestational age (*n*=113) and more than the required BMI (*N*=77) according to their gestational age were excluded. Only 64 preeclamptic primigravida were found eligible according to the study protocols, having low BMI for the gestational age; moreover, four participants refused to participate. Therefore, 60 underweight primigravida preeclamptic women were recruited in our study. They were allocated randomly (computer randomizer software version 3.0) into two groups 30 of them in placebo group and 30 in the LNS-PLW group as shown in Figure 1. On the trial day, two participants dropout one from LNS-PLW group who dislike the taste of supplement and other from the placebo group refuse to give the second blood sample after the consumption of supplement.

#### Study subjects and intervention

The objectives of the study were explained to every participant in their local language and a volunteer information sheet was provided to each participant. The information sheet included all the details of the study, including its purpose, inclusion/exclusion criteria, experimental tests required in our study, benefits and possible disadvantages of taking part in the study. The written informed consent was obtained after clarifying all queries of participants. The visual analogue scales (VASs) provided were also translated into local language for the convenience of the participants so that they could understand it well. Moreover, the participants were trained to mark it, prior to the main trial day at the time of recruitment by the main researcher. The participants were asked to come after 12 h of fasting to the nutritional trial room of Khyber Medical University or the side room of Matta Civil Hospital Swat Gynecology and Obstetrics department between 8:00 and 9:00 am for the measurement of energy intake, appetite and insulin and glucose concentration after the short-term consumption of the supplement.

**Figure 2:**
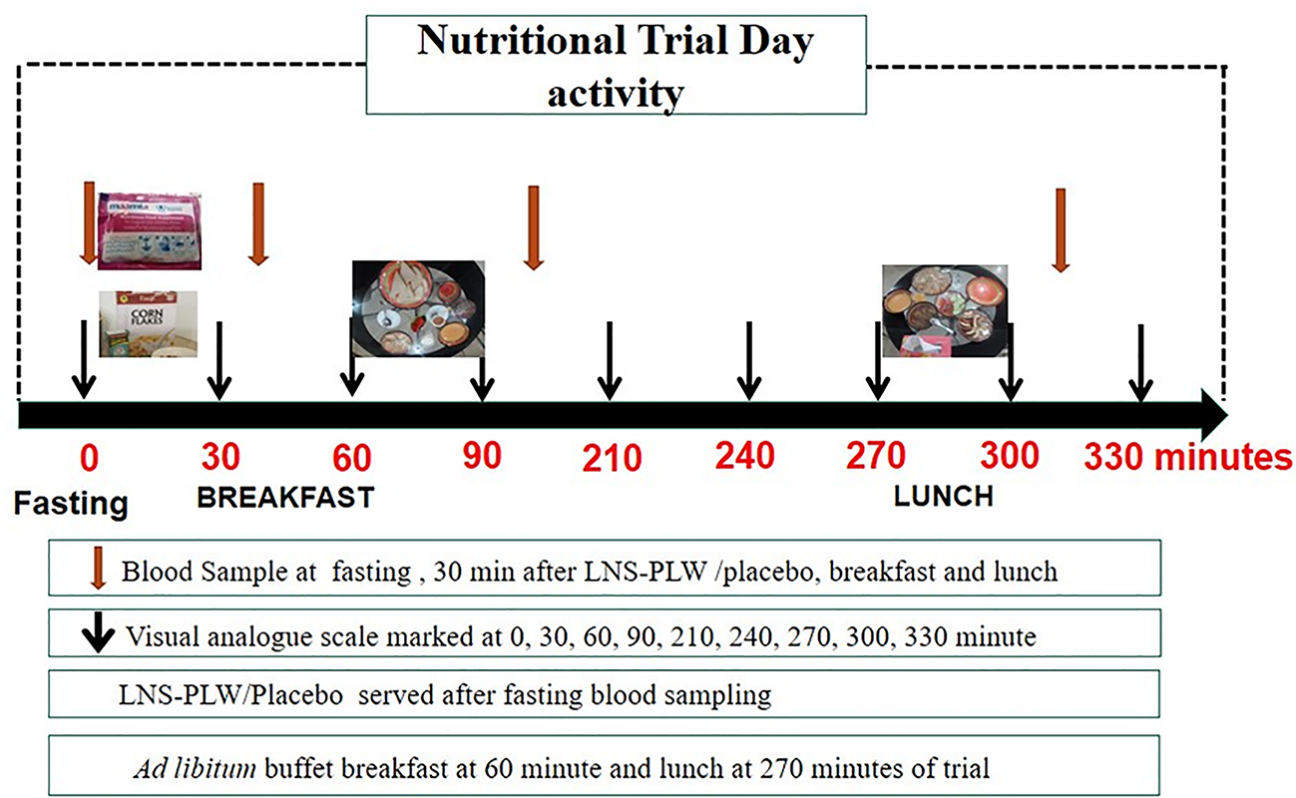
Study design for the RCT data collection points for appetite score, glucose and insulin levels on the day of trial

At arrival, women were allowed to rest and acclimatize to the clinic for 10 min. Then, intravenous cannula indwelling 18G was inserted in the antecubital vein of the forearm using an antiseptic technique. A fasting blood sample was collected. Then, the levels of appetite were evaluated using a validated VAS, which was marked as zero minute by participants (Figure 2).

A low-calorie supplement snack (75 g) containing skimmed milk (45 g) and Fauji cornflakes (30 g) was provided to the placebo group. The placebo used in the current study was selected after two pilot studies. Three placebo samples were prepared, in which the lowest caloric value (104 kcal/75 mg) was selected. The LNS-PLW group received 75 g of LNS supplement as shown in Table 1. The major nutrient composition of placebo and LNS-PLW were shown in Table 2. It was single-blind trial and the participants were not told what they would consume. The supplements were in the same colour, texture and consistency and were not distinguishable. The appetite scores were collected at 0, 30, 60, 90, 210, 240, 270, 300 and 330 min throughout the nutritional trial as shown in Figure 2.

**Table 2:** Nutrient composition of the supplements given to the placebo and LNS-PLW groups

	LNS-PLW (75 g)	Placebo (75 g)
Energy (Kcal)	400	104 kcal
Carbohydrate (g)	35.4	22.1
Protein (g)	10.5	2.2
Fat (g)	24	0.4
Carbohydrate energy	50.7%	89.4%
Protein energy	15.0%	6.1%
Fat energy	34.3%	4.5%

#### Appetite measurement

The VAS is a commonly used scale for the assessment of subjective sensations of appetite. It is used to evaluate the participant’s desire to eat, feeling of hunger, fullness, prospective food consumption and satiety [22–24]. The lines of VAS consist of 100 cm in length with words of feeling at the ends on both sides of the lines. The positive feelings are written on the right side of the scale and the negative feelings are on the left [22,24]. All the queries regarding the VAS application were cleared before the trial. The participants were requested to mark their feelings of satiety, hunger, fullness and desire to eat by a vertical line mark on the horizontal line [22]. The scores were computed by measuring the distance on the line from the left end [17,22]. To reduce the bias with the use of VAS are reporter bias in the interpretation of VAS. This was reduced as all the VAS were interpreted by the same researcher.

#### Ad libitum buffet meals

The ‘*ad libitum* buffet’ breakfast and lunch were prepared according to the choice of the subjects and were served after 1 and 4.5 h after supplement consumption. The energy intake during the trial was measured by using win diet software and reading was noted.

The ‘*ad libitum*’ buffet meals included a variety of uniform items with a total energy content that was around three times the participants’ estimated calorie requirements. The same setting and the same type of food were served at the same time to avoid any bias. The ‘*ad libitum*’ meals provided in both trials were the same, offering a variety of protein, fat and carbohydrates along with the same calorie and micronutrient contents. The participants had 30 min to finish their meal and were told to eat as much as they could comfortably eat based on their appetite. If needed, additional food and drink were always accessible during the trial. The breakfast included mixed milk black tea, sugar, skim milk, chickpea curry, jam, bread/nan and water. The lunch included water, apple/mango juice, chicken korma, savoury rice, chapattis, raw tomato, cucumber and apple. A low salt and oil-content diet were prepared. Before serving, the food item was divided into pieces to minimise portion-related cues. Before and after serving, every food item was weighed using an electric kitchen scale. The participants were unaware that the goal of the buffet meals was to measure their energy consumption [25,26].

#### 24-h dietary recall data

This tool was used to measure the habitual food intake of participants. This method entails inquiring, remembering, describing and measuring the consumption of food and drinks taken in the last 24 h before the nutritional trial day [27]. To reduced recall bias, a properly trained researcher collected the data after giving proper demonstration to participant. A serial of precisely asking describing and recalling method was used to measure all the drinks and food they ingested before participating in this study, from their first intake in the morning to their final meal or beverage before bed or when they got up at midnight. We utilized images of typical household dishes to measure the quantities and volumes. The purpose of these data collection was to obtain information of malnutrition, nutritional status and the dietary habits of the participants [27].

#### Measurement of nutrient composition and energy intake of study foods

All food and beverages were weighed with a digital kitchen scale. Food was not weighed in front of subjects to reduce potential bias and avoiding conscious eating. We calculated dietary energy (kcal) and macronutrient (carbohydrate, fat and protein) intakes by Windiet 2005 software [16,28]. To avoid any kind of error in results and ensure accuracy, the measurements were performed by three researchers independently and mean of the values of noted and taken as the final value for record.

#### Blood sampling

The blood samples were collected in an ethylenediamine tetra acetic acid EDTA tube from each participant on the days of the experimental trial under an aseptic technique. The blood was collected on nutritional trial day from each participant fasting at baseline on 0 min, 30 min after supplementation and after breakfast and lunch. Blood samples were centrifuged by 4000 rpm for 10–15 min in refrigerated centrifuge. The plasma supernatant was collected in Eppendorf tubes and stored at −80°C before analysis.

For insulin analysis the Abbott Architect c8000 system was used, utilizing potentiometric and photometric technology on immunoassay tests, utilizing CM1A (Chemiluminescent Microparticles assay). The glucose concentration was determined by enzymatic calorimetric method by using kits containing (glucose HK-CP reagent ABX Pentra, Horiba ABX, France) on the spectrophotometric analyser on automatic Roche Cobas Mira.

### Statistical analysis

The Shapiro–Wilk normality test was used for the determination of normality of data. The data were normally distributed. For comparing the blood levels and energy intake during trials paired *t-*test was used. For the assessment of the strength of the correlation between changes in appetite scores and changes in blood glucose and insulin levels, the regression slope and *R^2^* value for appetite scores on insulin and glucose levels at the certain time points were calculated. Student’s *t*-test was used for the comparison of regression slopes. Statistical analysis was performed with SPSS version 20. A *P*-value of 0.05 was used to establish significance in the statistical tests.

## Results

Sixty underweight preeclamptic primigravida were randomly and equally allocated into two groups: the LNS-PLW and the placebo. Two participants dropped, one refused to give the second blood sample after placebo and the other don’t like the supplement (LNS-PLW) taste. So, 58 participants completed the trial, 29 in each group for determining the effect on energy intake, insulin and glucose levels and appetite score (Figure 1).

### Characteristics of study patients

The participants of both groups were of same age (LNS-PLW: 23.30 ± 3.59 years; Placebo: 22.07 ± 3.34 years; *P*-value 0.174), and the BMI (kg/m^2^) at recruitment in LNS-PLW group was found to be low (LNS-PLW: 26.21 ± 3.39, Placebo: 27.99 ± 2.68, *P*-value: 0.04). While at recruitment, the systolic and diastolic blood pressure showed no significant difference as shown in Table 3.

**Table 3:** Anthropometric characteristic of the study groups

Characteristic	LNS-PLW (*n*=29); mean ± SD	Placebo (*n*=29); mean ± SD	*P*-value
Age of participants (years)	23.30 ± 3.59	22.07 ± 3.34	0.174
BMI (kg/m^2^) at the first antenatal visit from antenatal record	19.67 ± 0.85	19.61 ± 1.33	0.844
Gestational age at the first antenatal visit from antenatal record	20.5 ± 3.14	19.84 ± 3.5	0.450
BMI (kg/m^2^) at enrolment	26.21 ± 3.39	27.99 ± 2.68	0.038
Gestational age at enrolment	29.80 ± 2.31	30.10 ± 1.93	0.591
Systolic BP	144.67 ± 6.81	145.67 ± 8.98	0.629
Diastolic BP	95.50 ± 4.42	95.67 ± 5.20	0.894

### 24-h dietary recall

The 24-h dietary recall data were collected for measuring the habitual regular energy intake of the participants (Table 4).

**Table 4:** 24-h dietary recall data for study participants

	LNS-PLW; mean ± SD; *n*=29	Control; mean ± SD; *n*=29	*P*-value
Energy (kcal)	1716.93 ± 249.18	1719.59 ± 376.52	0.975
**Macronutrient**			
Fat (g)	65.16 ± 23.41	79.65 ± 22.54	0.310
Carbohydrate (g)	237.81 ± 40.81	250 ± 56.13	0.348
Protein (g)	61.60 ± 19.59	54.27 ± 13.49	0.102
**Micronutrient**			
Ca (mg)	665.48	694.24	0.644
Mg (mg)	258.31	246.45	0.444
Se (mcg)	30.90	22.69	0.178
Vitamin B_12_ (μg)	2.06 ± 1.69	2.52 ± 8.20	0.769
Folic acid (μg)	154.65 ± 63.15	152.86 ± 52.12	0.906
Fe (mg)	13.50 ± 4.08	15.31 ± 9.30	0.340

Abbreviations: Ca, calcium; Mg, magnesium; Se, selenium; Fe, iron; mean ± SD. Significant different (*P<0.05, **P>0.001).

### Energy intake data

**Figure 3:**
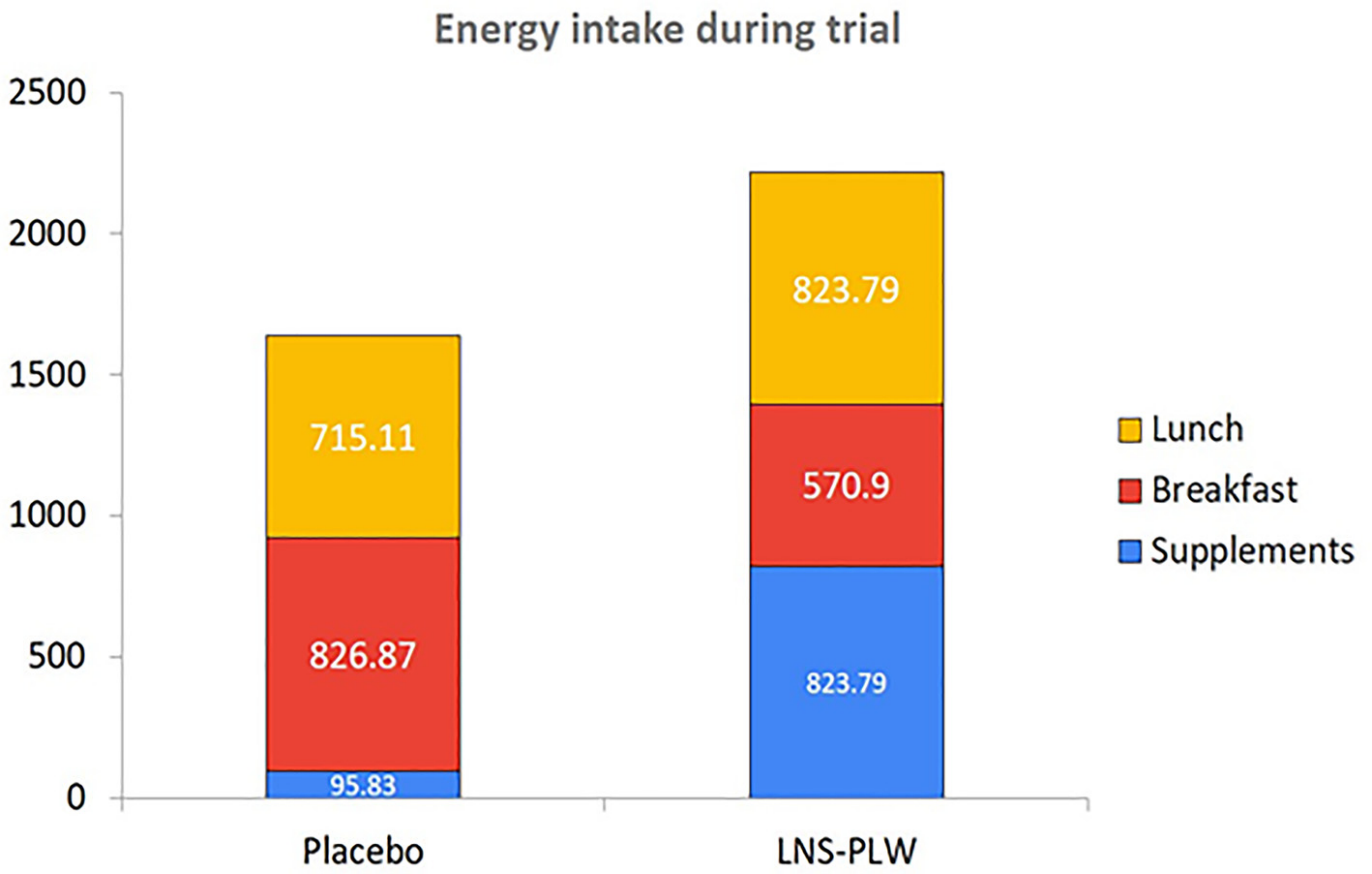
Energy intake during supplementation, breakfast and lunch in LNS-PLW/placebo trial

The breakfast energy intake was calculated, and a significant decline in the supplement ‘LNS-PLW’ group was observed (LNS-PLW: 570.90 ± 54.88 kcal; Placebo: 826.97 ± 175.21 kcal: *P*-value = 0.001). However, the energy intake at lunch time of LNS-PLW group increased significantly (LNS-PLW: 823.79 ± 68.04 kcal; Placebo: 715 ± 112.46 kcal: *P*-value = 0.004). The overall energy intake during the trial was significantly high in the ‘LNS-PLW’ group as compared with placebo (LNS-PLW: 1766.96 ± 78.22 kcal; Placebo: 1635.33 ± 211.14 kcal: *P*-value = 0.002) (Table 5 and Figure 3).

**Table 5:** Energy intake during LNS-PLW/placebo trial

		LNS-PLW; mean ± SD; *n*=29	Placebo; mean ± SD; *n*=29	*P*-value
LNS-PLW/Placebo (0 min of trial [after 12 h fasting] in morning)	Energy (kcal)	360.45 ± 26.26	95.83 ± 2.58	<0.001***
	Fat (g)	21.65 ± 1.57	0.4 ± 0.007	<0.001***
	Carbohydrate (g)	31.96 ± 2.32	20.46 ± 0.52	<0.001***
	Protein (g)	9.47 ± 0.70	2.09 ± 0.06	<0.001***
Breakfast (60 min of trial)	Energy (kcal)	570.90 ± 54.88	826.97 ± 175.21	<0.001***
	Fat (g)	13.73 ± 2.32	21.36 ± 5.14	<0.001***
	Carbohydrate (g)	95.48 ± 9.95	137.26 ± 29.51	<0.001***
	Protein (g)	22.08 ± 3.83	32.32 ± 6.07	<0.001***
Lunch (270 min of trial)	Energy (kcal)	823.79 ± 68.04	715 ± 112.46	0.004**
	Fat (g)	18.85 ± 1.31	16.59 ± 2.61	0.001**
	Carbohydrate (g)	130.14 ± 14.35	110.64 ± 21.53	0.001**
	Protein (g)	41.40 ± 3.27	36.26 ± 6.51	0.003**
LNS-PLW/Placebo, Breakfast, Lunch	Energy (kcal)	1766.97 ± 78.21	1634.34 ± 211.15	0.002**
	Fat (g)	54.50 ± 2.60	38.49 ± 5.38	<0.001***
	Carbohydrate (g)	261.41 ± 15.50	268.15 ± 38.23	0.382
	Protein (g)	73.33 ± 4.05	71.01 ± 8.42	0.185
				

Mean ± SD. Paired *t*-test done for comparison.

### Appetite responses

**Figure 4:**
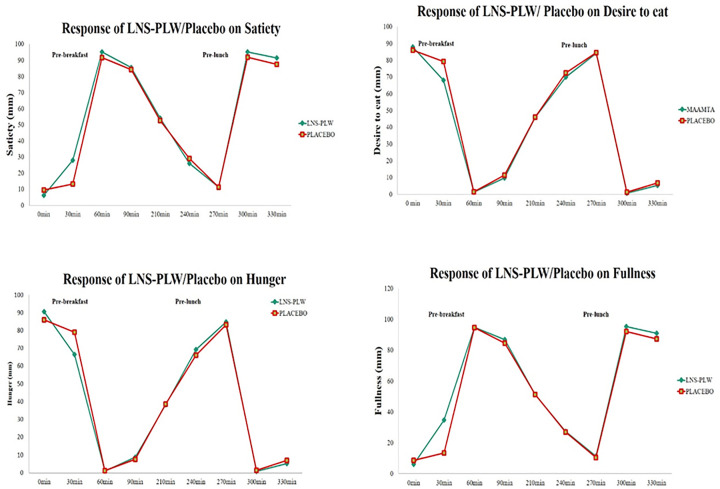
Measurements of satiety, desire to eat, hunger and fullness at fasted state (0 min), pre-breakfast (0–60 min) and pre-lunch (60–270 min) periods Values as mean ± SE are presented.

On the trial day during the fasting state, the appetite measure for the feeling of fullness, satiety, desire to eat and hunger were not different among both groups. However, during (0–60 min) the pre-breakfast duration, the VAS scores for the feeling of desire to eat was (LNS-PLW, 69 ± 21 mm; Placebo, 78 ± 17 mm, P=0.03) and the feeling of hunger was (LNS-PLW, 66 ± 5 mm; Placebo, 78 ± 4 mm, P=0.02). While the VAS scores of the feeling of fullness was (LNS-PLW, 36 ± 4 mm; Placebo, 14 ± 1 mm, P=0.00) and satiety VAS score was (LNS-PLW, 29 ± 3 mm; Placebo, 14 ± 1 mm, P=0.00).During (60–270 min) the pre-lunch duration, the VAS scores of the feeling of fullness (LNS-PLW, 12 ± 1 mm; Placebo, 12 ± 1 mm, *P*=0.67), satiety (LNS-PLW, 12 ± 1 mm; Placebo, 12 ± 2 mm, *P*=0.98), for the feeling of desire to eat (LNS-PLW, 85 ± 1 mm; Placebo, 85 ± 1 mm, *P*=0.85) and for the feeling of hunger (LNS-PLW, 85 ± 1 mm; Placebo, 84 ± 2 mm, *P*=0.41) were not significantly different (Figure 4).

### Impact of LNS-PLW on glucose and insulin levels

**Figure 5:**
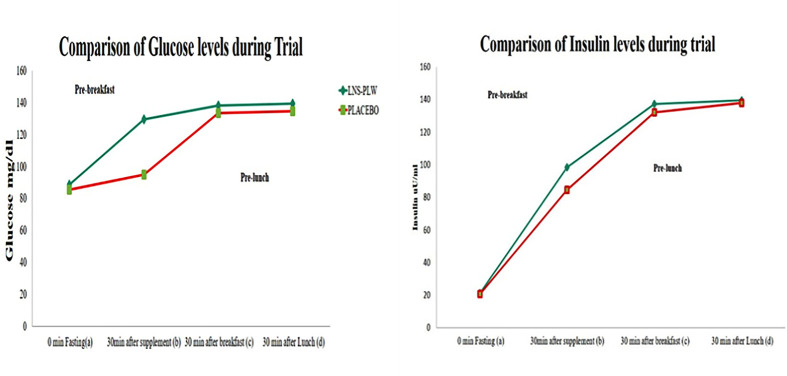
The comparison of glucose and Insulin concentration

At baseline, no significant difference was observed in fasting glucose levels of two groups. However, a significant increase was observed at 30 min after LNS-PLW consumption (LNS-PLW, 129.4 ± 5.4 mg/dL: Placebo, 94.7 ± 5.9 mg/dL, *P*<0.001). Likewise, 30 min after breakfast, glucose levels significantly inclined in the LNS-PLW consumers (LNS-PLW, 138.3 ± 3.9 mg/dL: Placebo, 133.4 ± 5.7 mg/dL, P = 0.001) as shown in Figure 5.

At baseline, the insulin levels showed no significant difference in both groups. However, 30 min after taking LNS-PLW, there was a significant increase in insulin levels (LNS-PLW, 98.5 ± 3.7 uU/ml: Placebo, 84.5 ± 2.7 uU/ml, *P*<0.001). Similarly, 30 min after taking breakfast, a significant difference between groups was noted (LNS-PLW, 137.3 ± 5.1 uU/ml: Placebo, 132.3 ± 6.5 uU/ml, *P*=0.002) as shown in Table 6 and Figure 5.

**Table 6:** Effects of supplementation on glucose and insulin concentrations

	Placebo; *n*=29	LNS-PLW; *n*=29	*P*-value
**Glucose (mg/dL)**			
Fasting (0 min)	85.7 ± 6.6	88.8 ± 5.5	0.060
30 min after supplement	94.8 ± 5.7	129.5 ± 5.2	0.001**
30 min after breakfast	133.3 ± 5.8	138.4 ± 3.8	0.001**
30 min after lunch	134.9 ± 4.9	139.4 ± 2.9	0.07
**Insulin (uU/ml)**			
Fasting (0 min)	20.6 ± 3.4	21.8 ± 2.7	0.150
30 min after supplement	84.5 ± 2.7	98.5 ± 3.7	0.001**
30 min after breakfast	132.3 ± 6.5	137.3 ± 5.1	0.002**
30 min after lunch	137.8 ± 5.3	139.5 ± 3.8	0.177

To assess the correlation strength between the changes in appetite measures and insulin and glucose concentrations, the regression slopes and *R^2^* value of appetite measures on particular time points are correlated with insulin and glucose concentrations. A Pearson’s correlation to find out the correlation and Student’s one-sample *t*-test was used for the comparison of regression slopes. There was no positive correlation between appetite scores and insulin and glucose concentrations as shown in Table 7.

**Table 7:** Correlation between appetite measures and glucose and insulin concentration

	Slope	95%CI	*R* ^2^	Correlation	*P*-value
**LNS-PLW**					
Fullness vs. Insulin	0.73 ± 0.18	-0.04, 1.50	0.89	0.94	0.06
Satiety vs. Insulin	0.72 ± 0.22	-0.21, 1.66	0.84	0.91	0.08
Desire to Eat vs. Insulin	-0.712 ± 0.22	-1.68, 0.25	0.83	-0.91	0.08
Hunger vs. Insulin	-0.739 ± 0.210	-1.64, 0.16	0.86	-0.92	0.07
**Placebo**					
Fullness vs. Glucose	1.56 ± 0.59	-0.99, 4.12	0.77	0.88	0.12
Satiety vs. Glucose	1.53 ± 0.68	-1.41, 4.46	0.71	0.85	0.16
Desire to Eat *vs*. Glucose	-1.49 ± 0.69	-4.49, 1.50	0.69	-0.82	0.16
Hunger vs. Glucose	-1.56 ± 0.67	-4.44, 1.31	0.73	-0.84	0.14

Mean ± SE, 95% CIs, mean of the slopes, *R*^2^ values and Pearson's correlation of the LNS-PLW group.

### Socioeconomic status

The socioeconomic data are tabulated in Table 8. The majority of the participants belongs to low-income groups. The literacy rate of the participants was also low with 76.7% of the placebo and 83.3% of the LNS-PLW group being found to be illiterate.

**Table 8:** The Socioeconomic data of study population

Parameters	Control; *n*=29	LNS-PLW; *n*=29
**Education**		
Uneducated	21 (72.41%)	20 (68.96%)
Middle	3 (10.34%)	5 (17.24%)
Matric	2 (6.90%)	2 (6.90%)
High school	1 (3.45%)	2 (6.90%)
Graduate	2 (6.90%)	-
**Occupation**		
Stay-at-home	28 (96.55%)	28 (96.55%)
Working	1 (3.45%)	1 (3.45%)
**Husband education**		
Uneducated	13 (44.80%)	13 (43.80%)
Middle	4 (13.80%)	2 (6.90%)
Matric	4 (13.80%)	7 (24.14%)
High school	2 (6.90%)	1 (3.45%)
Graduate	4 (13.80%)	5 (17.26%)
Master	2 (6.90%)	1 (3.45%)
**Husband profession**		
Labourer	9 (31.03%)	6 (20.69%)
Private Job	17 (58.63%)	15 (51.72%)
Jobless	1 (3.45%)	1 (3.45%)
Government Job	2 (6.89%)	7 (24.14%)
**Family income (PKR**)		
15,000–25,000	6 (20.69%)	3 (10.34%)
25,000–50,000	8 (27.59%)	10 (34.48%)
50,000–100,000	6 (20.69%)	11 (37.94%)
>100,000	9 (31.03%)	5 (17.24%)
**Family members**		
2–6	5	11
7–9	12	14
10–12	7	-
>12	5	4
**House structure**		
Pakka	14 (48.27%)	15 (51.73%)
Kacha	-	
Semi pakka	15 (51.73%)	14 (48.27%)
**Household**		
Isolated	5 (17.24%)	15 (51.73%)
Joint	24 (82.76%)	14 (48.27%)
**House type**		
City	10 (34.49%)	13 (44.83%)
Village	19 (65.51%)	16 (55.17%)
**No. of rooms**		
1–3	20	23
4–5	8	6
>5	1	-

The professional status showed that 96% of the participants were housewives. Most of the participants’ husbands were employees of private gas stations and shops, drivers and helpers, and were uneducated. The household status indicated that most of them lived in joint family, an average of 8 in the placebo group and 9 in the LNS-PLW group.

## Discussion

Lipid-based nutrition supplements (LNSs) are energy and nutrient-dense foods designed to prevent malnutrition among children and women in low-resource settings [29]. This study evaluates the response to LNS intake among underweight preeclamptic primigravida women in term of energy intake and appetite scores at breakfast and lunch as well as its impact on glucose and insulin levels.

### Effects of supplementation on energy intake

Initially, it was observed that LNS-PLW intake subsequently displaced food taken after it and suppressed the next meal, and ate much less at breakfast in comparison with the placebo group. Similar findings were observed by Fatima et al. [16] in which a decreased in energy intake resulted after the consumption of a nutrient-dense supplement drinks during the next meal. Comparable results have been reported in other studies [16,17,25,30]. Although the total energy intake by LNS-PLW/Placebo and breakfast when compared, it was noted that despite less food intake at breakfast the total intake of energy was significantly high in the LNS-PLW group. This indicates that a high intake of energy during LNS-PLW might compensate for less food consumption at breakfast. The current study results are similar to results reported by a few other nutritional trials in which high-energy supplement ingestion suppresses the next food intake [17,31,32].

It has also been observed that the compression of energy intake after LNS-PLW does not last long, and taking high-energy supplements in the early morning does not affect energy intake later during the day. In the current study, LNS-PLW users significantly increased their energy intake at lunch. The increased energy intake at lunch confirms that breakfast-time energy suppression in the LNS-PLW group is transient and short-lived. Several other studies have observed no suppression of energy intake at lunch in groups taking high-energy supplements [33,34]. Our findings contradict Fatima et al. but other studies found no significant difference in energy intake at lunch between groups. Their study population was underweight healthy women, they observed that fasting supplement intake in the morning prior to breakfast, decreased intake at breakfast but showed no impact on energy intake later on during the day [16,17]. This discrepancy may be due to the subject (non-pregnant women).

We observed that total energy intake during the trial was an average of 132.52 kcal high in the LNS-PLW trial than the Placebo trial. The total increase in the energy intake was less as compared to what was expected, the expected increase in energy intake was 360 kcal per day. Thus, our finding suggests that supplementation with multiple micronutrients nutritional supplement LNS-PLW provided to underweight preeclamptic primigravida enhances energy intake above daily energy requirement, but this increase in energy intake was less than expected. Our report is consistent with a study performed on undernourished healthy individuals suggesting only 17% more energy intake with supplementation [16]. Similarly, a study conducted on non-pregnant lean women reported a less than expected increase in total energy intake [16,17].

### Effects of supplementation on appetite scores

We also measured and compare the appetite scores during trials. We observed that increased satiety and suppressed hunger were more in the LNS-PLW group after 60 min of ingestion as compared with placebo ingestion.

These results are similar to studies conducted in young, slim women, who reported reduced hunger and increased satiety after high-energy dietary supplements (HENSDs) [16], while other studies contradicted our observations [35,36]. Similarly, the current study found that LNS-PLW increased satiety and decreased the desire to eat compared with placebo. These observations were consistent with the results of a study conducted in healthy, slim women [16], although the results of this study were conducted in non-obese, non-pregnant participants not consistent with another study [37]. The impact of the LNS-PLW/Placebo on appetite score after the breakfast till lunch (during pre-lunch period) was not different. This indicates that the suppressive effect of the LNS-PLW was short-lived. According to Stratton et al., bolus tube feeding of liquid high energy supplements to healthy person showed a slight impact on the VAS and little effect on the appetite measures. This difference of finding might be due to the study subjects. This is because participants’ nutritional requirements are higher due to pregnancy.

### Effects of supplementation on glucose and insulin levels

Glucose concentration after taking the LNS-PLW supplement was higher than those taking a placebo. Our results are parallel to the studies conducted on underweight subjects [16,19,38–41]. Insulin levels after taking LNS-PLW were also observed to be higher in the pre-breakfast period compared with the placebo. Our results were similar to some of the other supplementation trials [16,19,38–41]. Bellou et al. and Maraki et al. reported increases in both insulin and glucose concentrations after taking supplements [42,43] while Blundell et al. reported only increased insulin concentration, Lim et al. observed no changes in insulin and glucose and insulin concentrations after supplementation [44–46]. Insulin is reported an appetite suppressing effect, and high insulin concentration after consumption of LNS-PLW might cause displacement of subsequent meal [16]. An increase in satiety and decrease in the feeling of hunger 30–60 min after consuming of supplement can be attributed to higher levels of glucose and insulin [35]. The glucose and insulin both were thought to be appetite-suppressing hormones [16,39].

Although the energy intake of the LNS-PLW group was lower at breakfast but the blood glucose and insulin concentrations were found higher in the LNS-PLW group, there was a significant difference in the 60 min of post-breakfast blood glucose concentration. These post-breakfast rises in blood glucose indicate that the supplement compensated for the decrease in energy intake during breakfast. These findings contradicted by Fatima et al., they noted no difference between glucose levels significantly but significant increase in insulin concentrations was reported after the consumption of energy drinks and breakfast [16].

The suppressing effect on the appetite and increased glucose and insulin concentrations after LNS-PLW ingestion are evident from the less food intake at breakfast. It was noted that LNS-PLW ingestion induced a significant declined in hunger. Appetite responses after preload prior to breakfast are consistent with glucose and insulin concentration, and these findings are consistent with previous study in which the researchers administered high-energy liquid nutritional supplements [47]. The decreased hunger and increased satiety 60 min after supplementation were consistent with declined of energy intake from intake of breakfast, although the collectively the energy intake from the supplement and breakfast was significantly high in the LNS-PLW trial. The appetite score, insulin and glucose concentration and energy intake are in sequence with each other. However, post breakfast appetite score was similar in both trials, although the glucose levels elevated significantly 60 min post-breakfast in the LNS-PLW trial. Similarly, the LNS-PLW group showed a significant incline in insulin concentration after breakfast. These findings are consistent with the combined energy intake during LNS-PLW and breakfast when compared with the combined energy intake during the placebo and breakfast. This discrepancy among insulin and glucose concentration and appetite measurements has been contradicted by several studies [46,48] but our results are similar to others [16,49]. This difference may be due to their subjects who were normal lean healthy women while the added need of our pregnant subjects is the main confounding factor of our study.

### Correlation of appetite score and glucose and insulin levels

To further understand the relationship among the appetite scores, glucose and insulin responses, correlations were performed [16,50]. Correlations were performed within-group between the appetite measures and insulin and glucose levels. Also, the regression slope and R2 values of appetite score to insulin and glucose levels were calculated. It was noted that no significant correlation within-subject between appetite score and insulin and glucose responses was found. These findings, therefore, support the notion that appetite measurements are more correlated with energy intake than with insulin or glucose levels. These findings are consistent to Fatima et al. [16].

In short, intake of high-energy supplement LNS-PLW before breakfast reduces the feeling of desire to eat and feeling of hunger and increases feeling of fullness and satiety and simultaneously increases insulin and glucose concentrations, all of these suppress appetite. Increased feeling of satiety leads to reduced intake at breakfast might be due to the partial compensation of energy intake by LNS-PLW consumption. The overall energy provided in the LNS-PLW trial was not significantly higher than in the placebo trial. Daily consumption of LNS-PLW along with a normal diet, may improve nutritional status in underweight pre-eclamptic primigravida. Therefore, further studies are needed to investigate the partial compensatory mechanisms of energy intake provided by LNS-PLW consumption.

## Limitations

First, the participants of both groups were preeclamptic and no women with normal pregnancies were recruited for comparison. Second, we evaluate the short-term effects of LNS-PLW during strict laboratory conditions, which may not be representative of the clinical setting in routinely prescribed. Because the individuals’ typical eating habits may be affected by the experimental environment [16]. In addition, participants were sedentary most of the time during the trial, contrary to their normal lifestyle. Participants reported their appetite responses; therefore, the subject’s capacity to provide accurate information on feelings of satiety, fullness, hunger, and desire to eat completely determines the accuracy of self-reported replies on VAS. Mechanisms of energy suppression were not investigated, nor were appetite-regulating hormones measured, which was outside the scope of our study. Future research should be conducted.

## Conclusions

The consumption of LNS-PLW before the breakfast reduces the feeling of desire to eat and feeling of hunger and increases the feeling of fullness and satiety, and simultaneously increases insulin and glucose concentrations, all of which suppress appetite. Increased feeling of satiety leads to reduced intake at breakfast might be due to the partial compensation of energy intake by LNS-PLW consumption. The overall energy provided in the LNS-PLW trial was not significantly higher than in the placebo trial. Daily consumption of LNS-PLW along with a normal diet, may improve nutritional status in underweight pre-eclamptic primigravida. Therefore, further studies are needed to investigate the partial compensatory mechanisms of energy intake provided by LNS-PLW consumption.

The results may be only applicable to the underweight pre-eclamptic women for generalization after future studies with normal underweight pregnant women as control in multiple centres with large sample size and provision of longer duration of supplementation. Therefore, a multicentre trial, with large number of participants with a longer duration of supplementation is recommended.

## Institutional Review Board Statement

The study was conducted in accordance with the Declaration of Helsinki, and the study was approved by the trial institutional number (DIR/KMU-AS&RB/EN/000527) and ethical approval number (DIR/KMU = EB/EN/000314) from the Khyber Medical University Peshawar on 27th October 2016. And The **ISRCTN UK number** (ISRCTN15485068, April 2018: https://doi.org/10.1186/ISRCTN15485068).

## Informed Consent Statement

Informed consent was obtained from all subjects involved in the study.

## Data Availability

The authors will make the raw data supporting the conclusions of this article available, without undue reservation, to any qualified researcher.

## Abbreviations

LNS: lipid-based nutrition supplement
VAS: visual analog scale
WFP: World Food Programme
